# Introduction of Aromatic Ring-Containing Substituents in Cyclic Nucleotides Is Associated with Inhibition of Toxin Uptake by the Hepatocyte Transporters OATP 1B1 and 1B3

**DOI:** 10.1371/journal.pone.0094926

**Published:** 2014-04-16

**Authors:** Lars Herfindal, Camilla Krakstad, Lene Myhren, Hanne Hagland, Reidun Kopperud, Knut Teigen, Frank Schwede, Rune Kleppe, Stein Ove Døskeland

**Affiliations:** 1 Department of Biomedicine, University of Bergen, Bergen, Norway; 2 Translational Signaling Group, Haukeland University Hospital, Bergen, Norway; 3 BIOLOG Life Science Institute, Bremen, Germany; University of North Dakota, United States of America

## Abstract

Analogs of the cyclic nucleotides cAMP and cGMP have been extensively used to mimic or modulate cellular events mediated by protein kinase A (PKA), Exchange protein directly activated by cAMP (Epac), or protein kinase G (PKG). We report here that some of the most commonly used cyclic nucleotide analogs inhibit transmembrane transport mediated by the liver specific organic anion transporter peptides OATP1B1 and OATP1B3, unrelated to actions on Epac, PKA or PKG. Several cAMP analogs, particularly with 8-pCPT-substitution, inhibited nodularin (Nod) induced primary rat hepatocyte apoptosis. Inhibition was not mediated by PKA or Epac, since increased endogenous cAMP, and some strong PKA- or Epac-activating analogs failed to protect cells against Nod induced apoptosis. The cAMP analogs inhibiting Nod induced hepatocyte apoptosis also reduced accumulation of radiolabeled Nod or cholic acid in primary rat hepatocytes. They also inhibited Nod induced apoptosis in HEK293 cells with enforced expression of OATP1B1 or 1B3, responsible for Nod transport into cells. Similar results were found with adenosine analogs, disconnecting the inhibitory effect of certain cAMP analogs from PKA or Epac. The most potent inhibitors were 8-pCPT-6-Phe-cAMP and 8-pCPT-2′-O-Me-cAMP, whereas analogs like 6-MB-cAMP or 8-Br-cAMP did not inhibit Nod uptake. This suggests that the addition of aromatic ring-containing substituents like the chloro-phenyl-thio group to the purines of cyclic nucleotides increases their ability to inhibit the OATP-mediated transport. Taken together, our data show that aromatic ring substituents can add unwanted effects to cyclic nucleotides, and that such nucleotide analogs must be used with care, particularly when working with cells expressing OATP1B1/1B3, like hepatocytes, or intact animals where hepatic metabolism can be an issue, as well as certain cancer cells. On the other hand, cAMP analogs with substituents like bromo, monobutyryl were non-inhibitory, and could be considered an alternative when working with cells expressing OATP1 family members.

## Introduction

Analogs of cAMP with increased phosphodiesterase resistance, enhanced lipophilicity or specificity towards either of the cAMP-binding proteins cAMP-dependent protein kinase (PKA) and exchange protein directly activated by cAMP (Epac) have been extensively used to study the action of these proteins both in enzyme assays and in intact cells [1], [2], [3]. These analogs are considered selective to the cAMP-binding proteins, with few side effects, even from metabolites. One of the most used modifications of cAMP is the introduction of a thiophenyl-substituent at the 8-postion of the purine ring of cAMP. The corresponding analog 8-pCPT-cAMP is, when added extracellularly, a potent stimulator of cellular cAMP actions in intact cells, whether mediated by cAMP-dependent protein kinases, or the Epac class of cAMP receptors. However, recent studies have shown that metabolites of 8-pCPT-modified cAMP-analogs or the analogs themselves can affect signaling pathways or systems not regulated by cAMP-binding proteins, such as cortisol synthesis [4], [5], or binding of ligands to purinergic adenosine receptors on platelets [6], [7]. It is important to unveil such unspecific effects to ensure proper interpretation of cellular effects based on the use of nucleotide analogs.

The protein phosphatase (PP) inhibitors microcystin (MC) and nodularin (Nod) induce liver and intestinal failure and are considered a health hazard to humans if consumed in drinking water [8]. Uptake of MC and Nod is through liver specific organic anion-transporting polypeptides (OATP) [9], [10] and cause intracellular inhibition of protein phosphatase 1 (PP1) and PP2, generation of reactive oxygen species (ROS), and ultimately leads to apoptosis [11], [12], [13]. We have previously reported that Nod induced apoptosis is dependent on Ca^2+^/Calmodulin dependent protein Kinase II (CaMKII) [14], [15] and CaMKII inhibitors also potently inhibits Nod induced death. We wanted to find a possible role of cAMP signaling in Nod induced hepatocyte apoptosis. It is already known that cAMP induces cell death alone in leukemia cells [16], [17], and can modulate cell death induced by apoptosis inducers like Fas, TNFα, or anti-cancer drugs [18], [19]. However, we found effects of cAMP analogs that could not be ascribed to either activation or inhibition of PKA or Epac.

OATP1B1/1B3 are responsible for the hepatocellular uptake of MC and Nod [9], [20], and we have recently shown that inhibition of MC or Nod induced apoptosis may occur by inhibition of toxin uptake by OATP1B1 and 1B3 [20]. In that study, we also demonstrated a direct relationship between [^3^H]-Nod uptake and the cell's ability to undergo apoptosis. It is thus possible to measure OATP1B1 or 1B3 activity as a function of apoptosis induction [20]. OATP1B1 and 1B3 belong to the OATP1 subfamily and are predominantly expressed in the liver under normal conditions [21], [22], but can also be expressed in some solid cancers [23], [24], [25]. These proteins are responsible for uptake of several endogenous substances and drugs, and play a pivotal role in clearance of drugs from the blood stream (for a review, see [26]). Due to their substrate promiscuity [27], it is likely that OATP1B1/1B3 also interact with other compounds used both in the clinic and biomedical research [26]. If currently used cAMP analogs interfere with transport by these proteins they can alter the transport of other agents and themselves be subject to uptake inhibition by drugs or metabolites competing for transport into hepatocytes and also in cancer cells expressing these transporters.

MC and Nod are unable to penetrate biological membranes even if charged moieties are neutralized [28], but induce rapid apoptotic cell death if microinjected into non-hepatocyte cells [13]. Our findings show that modification of cyclic nucleotides can alter their substrate specificity to act on completely unrelated proteins. We report here that analogs of cAMP, cGMP or adenosine, with aromatic ring substituents inhibited OATP1B1/1B3 mediated transport without any correlation with their ability to modulate PKA or Epac activity. Some of these analogs are among the most commonly used to mimic Epac and/or PKA activation. It is also likely that aromatic ring substituents on other cyclic nucleotide analogs than those tested here can also affect transport mediated by OATP1B1/1B3. We call for caution when using such analogs and suggest using combinations of several analogs before concluding on effects mediated by PKA, Epac, or other enzymes modulated by cyclic nucleotides.

## Materials and Methods

### Ethics statement

The experiments including isolation of rat hepatocytes were approved by the Norwegian Animal Research Authority and conducted according to the European Convention for the Protection of Vertebrates Used for Scientific Purposes.

### Chemicals

The cyclic nucleotide analogs used in this study were: 8-(4-Chlorophenylthio)adenosine-3′, 5′-cyclic monophosphate (8-pCPT-cAMP), 8-(4-Chlorophenylthio)-2′-O-methyladenosine-3′, 5′-cyclic monophosphate, acetoxymethyl ester (8-pCPT-2′-O-Me-cAMP-AM), 8-(4-Chlorophenylthio)adenosine- 2′-deoxyadenosine-3′, 5′-cyclic monophosphate (8-pCPT-dcAMP), 8-(4-Chlorophenylthio)-2′-O-methyladenosine-3′, 5′-cyclic monophosphate (8-pCPT-2′-O-Me-cAMP), 8-(4-Chlorophenylthio)-2′-O-methyladenosine-3′, 5′-cyclic monophosphorothioate, Sp- isomer (Sp-8-pCPT-2′-O-Me-cAMPS), 8-(4-Chlorophenylthio)-2′-O-methyladenosine-3′, 5′-cyclic monophosphorothioate, Rp-isomer (Rp-8-pCPT-2′-O-Me-cAMPS), 8-(4-Chlorophenylthio)-N^6^-phenyladenosine-3′, 5′-cyclic monophosphate (8-pCPT-6-Phe-cAMP), 8-(4-Chlorophenylthio)-N^6^-phenyladenosine-2′-deoxyadenosine 3′, 5′-cyclic monophosphate (8-pCPT-6-Phe-dcAMP), 8-Bromoadenosine-3′, 5′-cyclic monophosphate (8-Br-cAMP), 8-Bromoadenosine-3′, 5′-cyclic monophosphorothioate, Rp-isomer (Rp-8-Br-cAMPS), 8-Bromo-2′-O-methyladenosine-3′, 5′-cyclic monophosphate (8-Br-2′-O-Me-cAMP), N^6^-Monobutyryladenosine-3′, 5′-cyclic monophosphate (6-MB-cAMP), 8-(4-Chlorophenylthio)guanosine-3′, 5′-cyclic monophosphate (8-pCPT-cGMP), 8-Bromoguanosine-3′, 5′-cyclic monophosphate (8-Br-cGMP), 8-(4-Chlorophenylthio)adenosine (8-pCPT-Ado), 8-(4-Chlorophenylthio)-2′-O-methyladenosine (8-pCPT-2′-O-Me-Ado), N^6^, 2′-O-Dibutyryladenosine-3′, 5′-cyclic monophosphate (DB-cAMP, Bucladesine). All cAMP, cGMP and Ado analogs were from Biolog LSI (Bremen, Germany). Nod was isolated from the cyanobacterium *Nodularia spumigena* strain AV1 as described [28], and labeled with [^3^H] by reduction with sodium boro[^3^H]hydride (Amersham Biosciences, Little Chalfont, U.K.) by the method given in [29]. [^14^C]-Glycocholic acid was from Amersham Biosciences, and Rifamycin SV, adenosine, cAMP and cGMP were from Sigma-Aldrich (St. Louis, MO, USA).

### Cell handling and experimental conditions

Primary rat hepatocytes were isolated from male Wistar rats by in vitro collagenase perfusion [30], [31]. Isolated hepatocytes were suspended (1.2×10^6^ cells/ml) in pregassed (95% O_2_/5% CO_2_) low-phosphate incubation buffer (10 mM Hepes, pH 7.4, with 120 mM NaCl, 5.3 mM KCl, 0.01 mM KH_2_PO_4_, 1.2 mM MgSO_4_, 1.0 mM CaCl_2_, 5 mM lactate, 5 mM pyruvate, and 0.5% bovine serum albumin) and allowed to condition in an incubator (37 °C, 5% CO_2_, humidified atmosphere) for 20 min prior to, and during the experiments. The cells were seeded in 48-well tissue culture dishes, and analogs or vehicle added with or without Nodularin (Nod). For microinjection, cells were seeded in gridded petri dishes. Insulin (0.2 nM) and dexamethasone (5 nM) was added after two hours of culture to maintain the metabolic competence of the cultured hepatocytes, and EGF (9 nM) 20 hours after seeding to stimulate growth. Cells received cytoplasmic injections of PKA subunit (RI_D199_) dissolved in 93 mM K-PO_4_/14 m mM Na-PO_4_ buffer (pH 7.2) containing 3 mM MgSO_4_, 1 mM gluthatione and 0.3 mM ATP.

Human embryonic kidney cells (HEK293T, ATTC #: CRL-11268) were cultured in Dulbecco's modified eagle medium (DMEM) supplemented with 10% fetal bovine serum (both from Invitrogen, Carlsbad, CA) and antibiotics (50 U/mL of penicillin and 0.05 mg/mL streptomycin). Enforced expression of green fluorescent protein (GFP) alone, or with OATP1B3 or OATP1B1 was obtained by calcium phosphate transfection of cells for 6 h. Vectors for OATP1B1 [22], and 1B3 [21] were a gift from Dietrich Keppler, German Cancer Research Center, Division of Tumour Biochemistry, Heidelberg, Germany.

Measurements of intracellular cAMP levels in un-transfected HEK293T cells were conducted as previously described [32]. Estimation of sodium fluorescein accumulation in OATP1B1/1B3 transfected cells was as described by [33], except that the cells were seeded in 24-well tissue culture plates at a density of 200 000 cells/well. Inhibitors were added 10 min prior to addition of sodium fluorescein, and the cells were then co-incubated with inhibitor and substrate for three min before removal of medium and washing.

### Immunofluorescence staining and confocal microscopy

HEK293T cells with enforced expression of OATP1B3 cultured on cover slips were fixed in 2% paraformaldehyde and permeabilized using 0.1% Triton X-100 in PBS. The samples were blocked in PBS containing 1 mg/ml BSA and 0.05% Triton X-100. Visualization of OATP1B3 was by immune-staining using rabbit anti-OATP-8, (Santa Cruz Biotechnologies, Santa Cruz, CA) and a FITC-conjugated goat anti-rabbit secondary antibody. Actin was visualized by fluorescein-conjugated phalloidin (F432, Molecular Probes, Leiden, Netherlands), and DNA by Hoechst 33342, and images were acquired by a Zeiss LSM 510 META confocal laser microscope fitted with a 40x/1.3 NA Plan-Neofluor oil-immersion objective (Zeiss, Oberkochen, Germany).

### Determination of hepatocyte uptake of radiolabeled nodularin and clycocholic acid

Freshly prepared primary rat hepatocytes in suspension (800 000 cells/ml) were incubated in buffer with cyclic nucleotide analogs, sulfobromophthalein (BSP) or vehicle for 15 min. Radiolabeled [^3^H]-Nod was added and the cells were incubated for another 5 min before rapid separation from the medium by centrifugation through a layer of a mixture of dibutyl and dinonyl phthalate [20], [34]. The cell pellet was lysed in 1 ml of 2% sodium dodecyl sulfate (SDS) for 1 h and the radioactivity determined by scintillation counting (TriCarb 3100TR, Perkin Elmer, Waltham, MA). The accumulation of [^14^C]-glycocholic acid was determined similarly, except that the cells were separated from the medium by gentle filtration (0.45 µm HA filters, Millipore, Billerica, MA). The filters, containing the cells, were washed twice with 2 ml of incubation medium (37 °C), and processed for scintillation counting (TriCarb 3100TR) using Emulsifier-Safe™ (Perkin Elmer, Waltham MA) to dissolve the cells. We found that 50 µM of BSP was sufficient to obtain complete inhibition of Nod or glycocholic acid uptake.

### Estimation of IC50 values of cyclic nucleotide analogs

Experiments were conducted 48-well tissue culture plates (35 000 cells/well). Every cyclic nucleotide analog was tested for its ability to inhibit Nod-induced apoptosis in OATP1B1- or 1B3-transfected HEK293 cells with a dose curve from 1 to 300 µM. Analogs were added to the transfected HEK293T cells 15 min prior to addition of Nod, and the cells were thereafter incubated for another 90 min with both analog and Nod. For some analogs, medium without serum was used to avoid enzymatic degradation of the analogs. This did not affect the cell's response to Nod. Apoptosis was assessed by microscopic evaluation of surface budding of GFP positive cells [20] using a Zeiss Axiovert 35 M inverted fluorescence microscope (Carl Zeiss MicroImaging GMbH, Göttingen, Germany). In brief, cells responding to Nod detached, had polarized blebbing and condensed actin. For estimation of cell death, only the population of GFP-positive cells was used. The data was used to estimate IC50 values by four-parameter curve fitting using SigmaPlot software (Systat Software Inc. San Jose, CA):
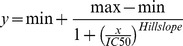
(1)

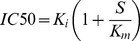
(2)


Nonlinear regression to estimate IC50 was performed using Eq. 1, where *y* is percent apoptotic cells observed, *min* is percent apoptosis in non-treated cells, *max* is the percent apoptosis in absence of inhibitor, at the given concentration of Nod, and *x* is concentration of inhibitor. At certain conditions the IC50 value is related to the competitive inhibitory constant (K_i_) of the transporter for the inhibitor x (Eq. 2), where S is the extracellular concentration of toxin accessible for the transporter with half maximal transport rate at S = K_m_.

We have previously described how competitive inhibitors of the nodularin transporters can be robustly assessed by a sensitive apoptosis assay [20]. A sigmoid function (i.e. a Hill equation) is used as the apoptosis process show a cooperative response curve, where the steepness depends on the cell type used and the time of measurements. In the case where only transport of the toxin determine the time of death of cells, the IC50 value of Eq. 1 would relate to the inhibitory constant (K_i_), however with different values depending on the level of toxin present (S) during the assay and of the affinity of the toxin for the transporter (K_m_, Eq. 2). These calculations are presented in Table S1.

The data for uptake of fluorescein into HEK293T cells transfected with OATP1B1 or OATP1B3 in the absence or presence of inhibitor were fitted in SigmaPlot, using Michaelis Menten kinetics with competitive inhibition. V_max_ and K_m_ values obtained in absence of inhibitor were used to fit the K_i_ value for uptake in the presence of inhibitor.

## Results

### cAMP analogs inhibit nodularin induced hepatocyte apoptosis independently of PKA or Epac activation

**Figure 1:**
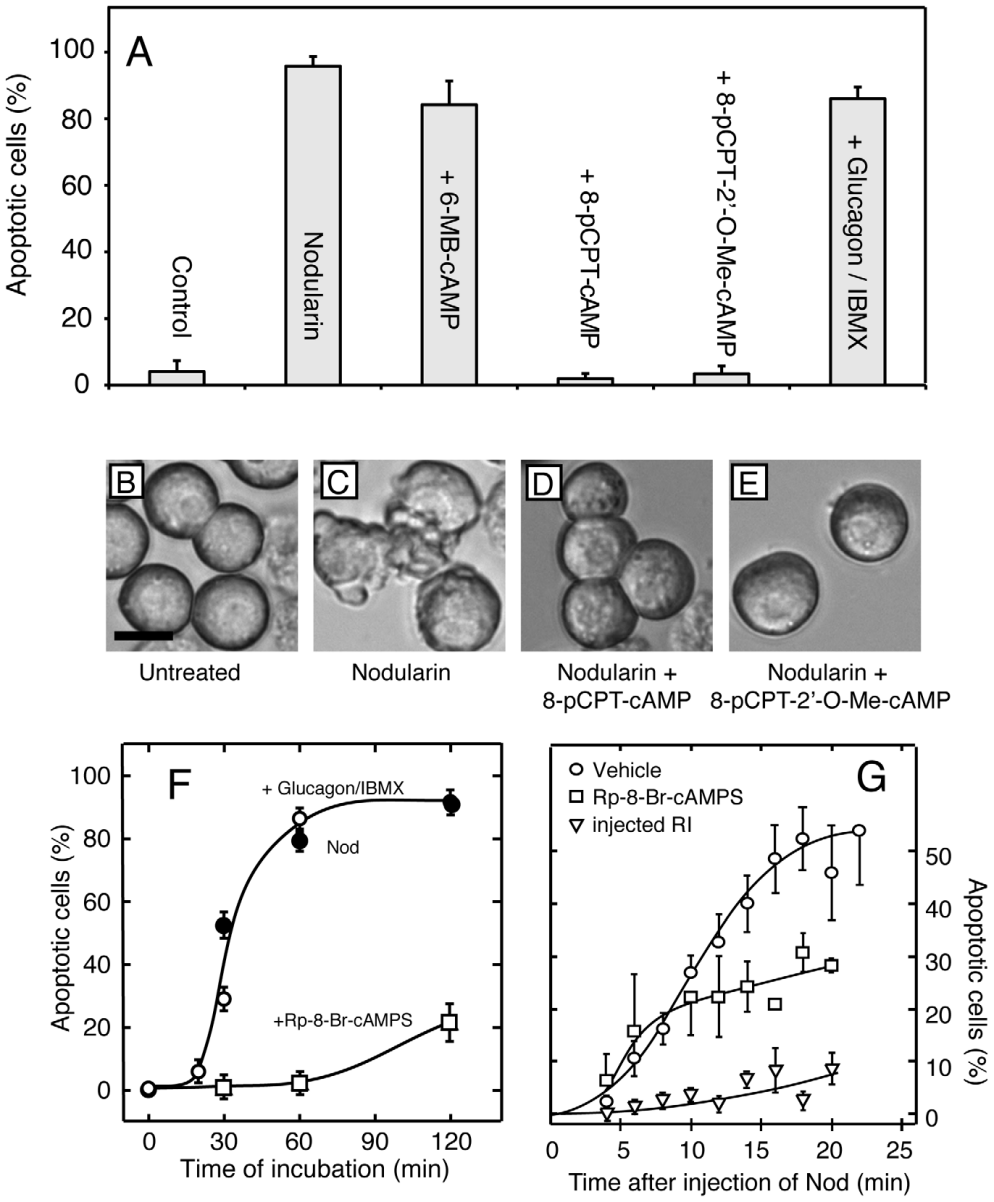
The ability of cAMP-analogs to modulate Nod-induced apoptosis is not related to the cAMP activated proteins PKA or Epac. A: Freshly isolated hepatocytes were incubated with the given analogues or vehicle for 15 min before addition of 150 nM nodularin. The cells were incubated for another 60 min before fixation in 2% buffered formaldehyde, and apoptosis scored by microscopic evaluation of surface morphology. The data are average of 3–5 experiments and SEM. B–E: Surface morphology of normal hepatocytes (B), hepatocytes treated with 150 nM Nod alone (C), hepatocytes treated with 150 nM Nod and 8-pCPT-cAMP (D) or 8-pCPT-2′-O-Me-cAMP (E) 15 min before addition of Nod. Bar represent 20 µm. F: Hepatocytes were treated as in A, but given the 300 µM of the PKA inhibitor Rp-8-Br-cAMPS. G: Primary hepatocytes were cultured for three days before microinjection with vehicle, Rp-8-Br-cAMPS or the negatively dominant mutant PKA R-subunit RI_D199_. One hour thereafter, the cells were microinjected with Nod to reach a final concentration of 2 µM inside the cells, and percent apoptosis was scored. The data represent the average of three to four experiment where 10-15 cells were microinjected, and SEM.

We first tested if cAMP analogs directed towards PKA, Epac or both enzymes could modulate nodularin (Nod) induced hepatocyte apoptosis. Nod alone induced apoptotic morphology (Fig. 1A and C), and activation of PKA by 6-MB-cAMP gave no change in apoptosis induction (Fig. 1A). However, both the Epac activator 8-pCPT-2′-O-Me-cAMP and the non-selective analog 8-pCPT-cAMP inhibited completely apoptotic morphology in the primary hepatocytes (Fig. 1A, D and E). This was in contrast to the finding that elevation of intracellular cAMP by glucagon and IBMX [35] did not alter the Nod effect (Fig. 1A). To further probe the role of PKA in Nod-induced hepatocyte apoptosis, we added the PKA-inhibitor Rp-8-Br-cAMPS to the hepatocytes prior to addition of Nod. Rp-8-Br-cAMPS inhibited apoptotic morphology (Fig. 1F), and microinjection of the negative regulator of PKA, RI into hepatocytes was also a potent inhibitor of Nod-induced hepatocyte apoptosis.

**Figure 2:**
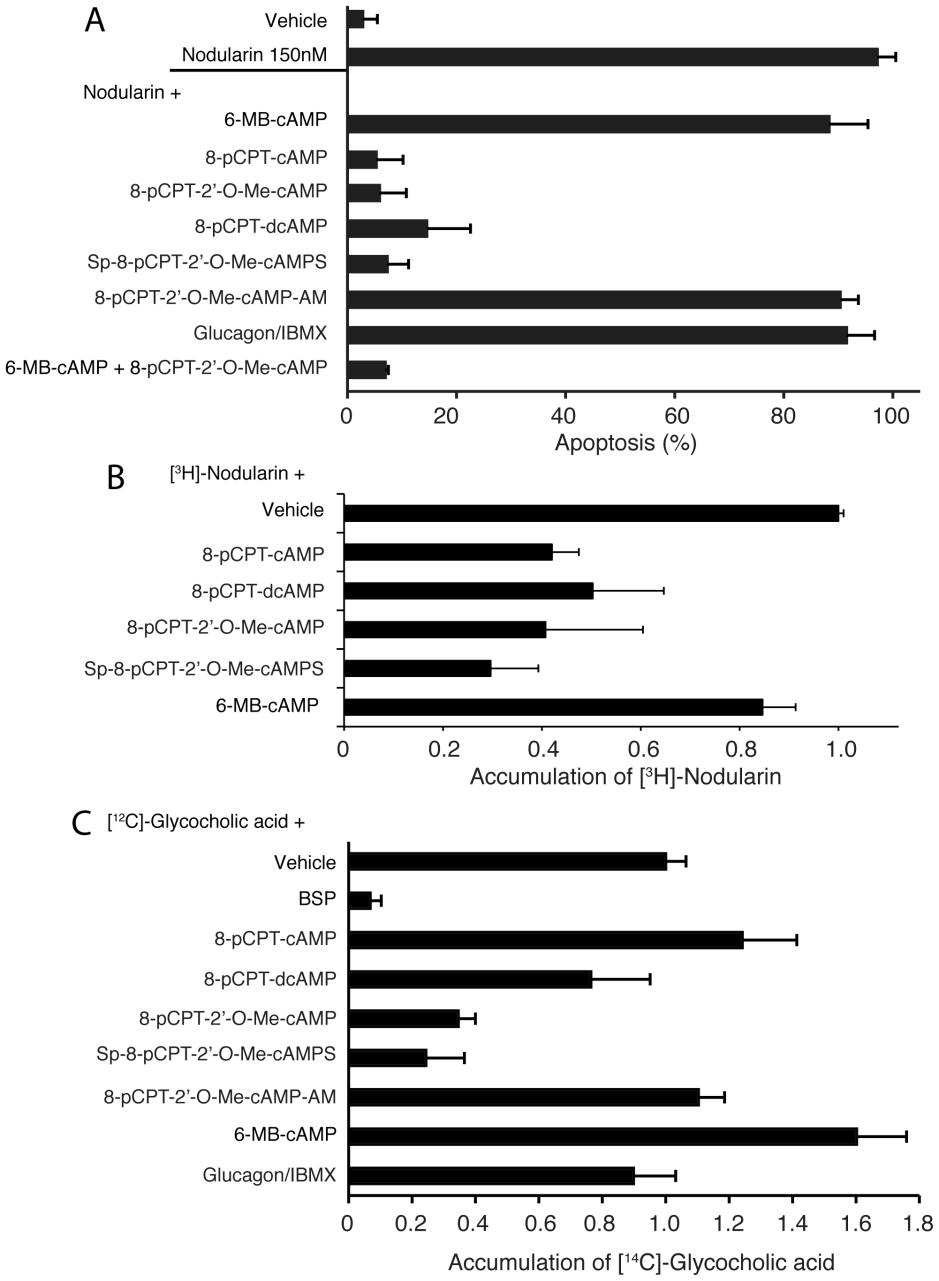
The ability of cAMP analogs to inhibit hepatocyte apoptosis coincides with inhibition of Nod and bile acid uptake. A: Freshly isolated hepatocytes were incubated with vehicle or cAMP analogs (50 µM) for 15 min before addition of 150 nM nodularin followed by co-incubation for another 60 min. The cells were next fixed and apoptosis scored as described in Figure 1. B: Freshly isolated hepatocytes were incubated with vehicle or cAMP analogs (50 µM) for 15 min before addition of [^3^H]-nodularin. The cells were incubated for another 5 min before separation of cells from supernatant and determination of intracellular radioactivity as described in the Experimental section. C: Freshly incubated hepatocytes were treated as in B, but added [^14^C]-glycocholic acid instead of nodularin. Cells were separated from supernatant by gentle filtration and intracellular radioactivity determined. The concentration of cAMP analogs in B and C is 50 µM. The data are average from 3-5 experiments and SEM.

To study the effect of various analogs in detail, we treated hepatocytes with different Epac and PKA selective cAMP analogs before addition of Nod. Again, we found clear inhibition with Epac activators such as 8-pCPT-cAMP, its 2′-O-Me analog and the thioate Sp-8-pCPT-2′-O-Me-cAMPS. Intriguingly, the 2′-deoxy variant 8-pCPT-dcAMP, which hardly affects PKA or Epac, also blocked Nod-induced apoptosis (Fig. 2A). We wanted to explore whether the lack of inhibition by endogenous elevation of cAMP by glucagon/IBMX (Fig. 1A and 2A) was due to activation of PKA. The addition of these agents increases endogenous levels of cAMP in hepatocytes [35] (see also Fig. S1 for the effect on HEK293-cells), and activates PKA. We added a combination of the PKA activator 6-MB-cAMP and the Epac activator 8-pCPT-2′-O-Me-cAMP. However, the combination of these analogs was an equally potent inhibitor of Nod-induced hepatocyte apoptosis as 8-pCPT-cAMP or 8-pCPT-2′-O-Me-cAMP alone (Fig 2A). Moreover, the membrane permeable, bioactivatable AM-ester [36] 8-pCPT-2′-O-Me-cAMP-AM did not inhibit Nod-induced apoptosis (Fig. 2A), excluding intracellular effects of the analogues, but rather indicating effects at the cell membrane.

We thus concluded that the analog's ability to inhibit Nod-induced apoptosis was not related to modulation of PKA and/or Epac.

### The ability of cAMP analogs to inhibit apoptosis coincides with inhibition of hepatocyte uptake of nodularin or bile acid

Nod and MC are internalized into hepatocytes by liver-specific broad substrate transporter proteins (OATP1B1 and 1B3 in human, Oatp1b2 in rat) [37]. We added radiolabeled MC or Nod to investigate if the inhibiting analogs decreased accumulation of toxins inside the primary hepatocytes. The same analogs that inhibited Nod-induced cell death (Fig. 1A and 2A) significantly decreased accumulation of [^3^H]-Nod (Fig. 2B). It thus appears that some cAMP analogs inhibit OATP1B1/1B3-mediated toxin transport into hepatocytes. These transporters can, together with others such as Na^+^-dependent taurocholate co-transporting polypeptide (NTCP), OATP1A2 and 2B1, also transport bile acids into hepatocytes [38], and we replaced [^3^H]-Nod with [^14^C]-glycocholic acid (GCA) to investigate if cAMP-analogs could inhibit a broad range of bile acid transporters. Sulphobromophthalein (BSP) was used to achieve full inhibition [20]. In the first set of experiments with [^14^C]-GCA, we were puzzled to find that 8-pCPT-cAMP did not inhibit accumulation, but caused a slight increase of [^14^C]-GCA inside the hepatocytes, similar to the PKA activator 6-MB-cAMP (Fig. 2C). However, the inactive 2′-deoxy variant 8-pCPT-dcAMP inhibited uptake, and as with [^3^H]-Nod (Fig. 2B), the Epac activator 8-pCPT-2′-O-Me-cAMP and Sp-8-pCPT-2′-O-Me-cAMPS almost completely inhibited [^14^C]-GCA accumulation (Fig. 2C). Again, we found no inhibitory effect of the membrane permeable Epac-agonist 8-pCPT-2′-O-Me-cAMP-AM (Fig. 2C). Surprisingly, we found an inverse dose-dependence relationship (Fig. S2) between PKA activation and [^14^C]-GCA accumulation, with increasing doses of either 8-pCPT-cAMP or 6-MB-cAMP leading to reduced accumulation. In fact 200 µM of 8-pCPT-cAMP led to accumulation of 60% compared to control. A similar trend was seen with 6-MB-cAMP where the accumulation of [^14^C]-GCA decreased from about 1.7 with 50 µM of to 1.1 (rel. to control) using 400 µM (Fig. S2). It thus appears that low concentrations of PKA activators could enhance accumulation of [^14^C]-GCA.

We conclude that there is a correlation between cAMP-analog's ability to inhibit Nod-induced hepatocyte apoptosis and to prevent accumulation of substrates of OATPs and other bile acid transporters.

### Linking common structural moieties with ability to inhibit OATP1B1 and 1B3

**Figure 3:**
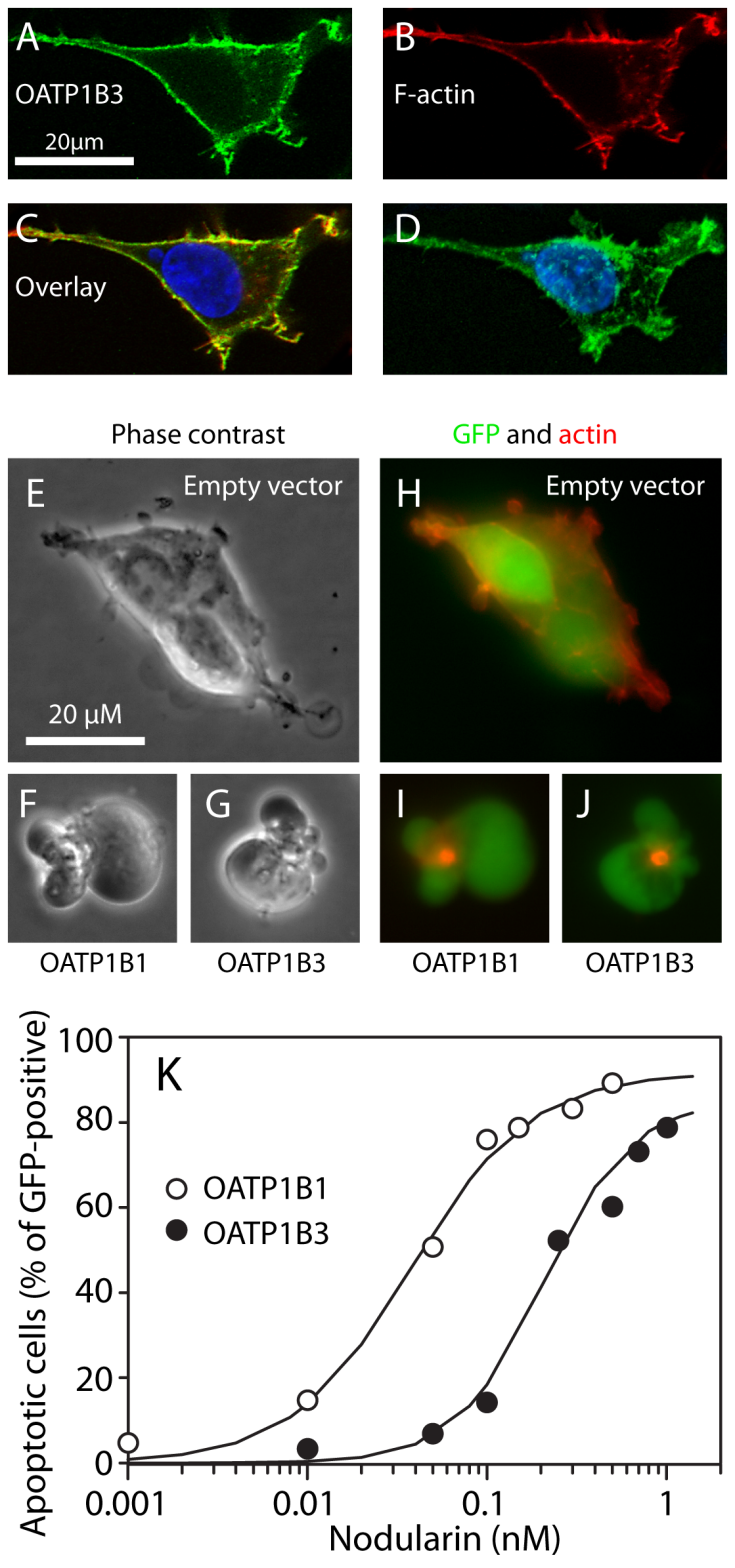
HEK-293T cells expressing OATP1B1/1B3 undergo apoptosis upon nodularin treatment. A-D: Confocal micrographs of a HEK-293T cell with enforced expression of OATP1B3. Visualization of OATP1B3 stained green (A), F-actin stained red (B), and composite image (C) of OATP1B3 (green), F-actin (red) and nucleus (blue). D: 3D composite micrograph showing cell membrane localization of OATP1B3. E-J: Phase contrast and fluorescent micrographs of HEK-293T cells co-transfected with GFP and empty vector (E, H), OATP1B1 (F, I) or -1B3 (G, J) treated with nodularin. After nodularin exposure for 90 minutes, the OATP-expressing cells showed distinct polarized budding (F, G) with hypercondensed actin (I, J), whereas cells treated with empty vector remained unaffected (E, H). K: HEK-293T cells expressing OATP1B1 or -1B3, were treated with various concentrations of Nod for 90 min before assessment of apoptosis based on surface morphology (E–G). Only GFP-positive cells were counted.

**Figure 4:**
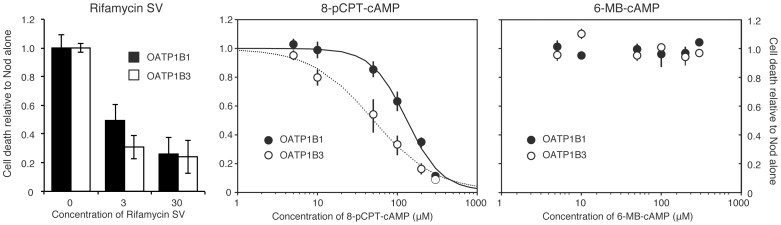
8-pCPT-cAMP inhibits Nod-mediated apoptosis in OATP1B1- or 1B3 transfected HEK293T cells. HEK293T cells were transfected with OATP1B1 or OATP1B3, and treated with the OATP1B1/1B3 inhibitor rifamycin SV, or the cAMP analogs 8-pCPT-cAMP or 6-MB-cAMP for 15 min before addition of Nod (0.1 µM for OATP1B1 and 0.6 µM for OATP1B3). After another 90 min incubation, the cells were fixed, and apoptosis assessed as described in the methods section. The results show cell death relative to that induced by Nod without cAMP analogs, and are mean and s.e.m. of 3-5 experiments.

Having disconnected the analog's PKA- and Epac-modulating effects from their ability to inhibit Nod-induced apoptosis, we next wanted to establish which structural moieties were responsible for the inhibition. We transfected HEK293T cells with OATP1B1 or 1B3, and studied their ability to undergo Nod-induced apoptosis with or without analogs present in the medium. The cells expressed the channels at the surface (Fig. 3A and D), close to the cortical actin (Fig. 3B and C). While cells transfected with empty vector remained unaffected upon Nod treatment (Fig. 3E and H), the cells transfected with either of the channels showed typical signs of Nod-induced apoptosis like detachment from the surface, polarized membrane blebbing, and relocalization and condensation of actin to the area of bleb origin [15] (Fig. 3F, G, I and J). The LC50 value of Nod was 50 nM for OATP1B1-expressing cells, and 220 nM for OATP1B3-expressing cells after 90 min incubation (Fig. 3K). The apoptogenic effect of Nod in the OATP1B1/1B3 transfected cells was abolished if the inhibitor rifamycin SV [39] (Fig. 4A) or 8-pCPT-2′-O-Me-cAMP (Fig. 4B) was added 15 prior to addition of Nod, whereas 6-MB-cAMP had no inhibitory effect (Fig. 4C). IBMX (250 µM) did not affect Nod-induced apoptosis in the OATP1B1/1B3 transfected cells (data not shown). We next tested whether 8-pCPT-2′-O-Me-cAMP also could inhibit the accumulation of the OATP1B1/1B3 substrate sodium fluorescein [33] into transfected HEK293T cells. The Km value for fluorescein uptake in our system was 402±140 µM for OATP1B1 and 285±134 µM for OATP1B3 (± s.e. of the estimate). The regression analysis showed that 8-pCPT-2′-O-Me-cAMP inhibited sodium fluorescein accumulation in a competitive manner with Ki value of 46±21 µM for OATP1B1 and 75±22 µM for OATP1B3 (R^2^ values for regressions, 0.79 and 0.91 respectively). We concluded that the nodularin based method and fluorescein method both showed potent inhibition of OATP1B1/1B3 substrates by 8-pCPT-2′-O-Me-cAMP. Although more complex and cumbersome, the Nod based method had the advantage that the background apoptosis was negligible (less than 5% in non-transfected cells).

We next incubated OATP1B1- or 1B3-expressing HEK293T cells with various concentrations of cAMP analogs and calculated their IC50 value towards Nod-induced apoptosis (Figure 4 and Table 1). The incubation time with analogs was 15 min, which is sufficient to modulate activity of PKA in blood platelets [6] and cardiomyocytes [40], or Epac in endothelial cells [41], to reveal a role of these enzymes in Nod-mediated apoptosis in HEK293T cells. We found that all cAMP analogs containing the 8-pCPT-moiety were potent inhibitors of OATP1B1/1B3 mediated Nod transport. These analogs were more potent inhibitors of OATP1B3 than 1B1. Noteworthy, the addition of a 2′-O-Me-moiety significantly increased the ability to inhibit transport through both channels, whereas the 2′-deoxy variant 8-pCPT-dcAMP, which hardly affects PKA or Epac, was only slightly less potent than the PKA/Epac activator 8-pCPT-cAMP.

**Table 1:** Ability of various nucleoside analogues to inhibit HEK293T-cell apoptosis induced by nodularin^1^.

		OATP1B1		OATP1B3	
	Analog	IC50	R^2^	IC50	R^2^
	cAMP	>300	–	>300	–
pCPT-substituted cAMP analogs	8-pCPT-cAMP	130	0.98	49.6	0.98
	8-pCPT-dcAMP	223	0.94	55.7	0.98
	8-pCPT-2′-O-Me-cAMP	46.4	0.98	12.6	0.96
	Sp-8-pCPT-2′-O-Me-cAMPS	19.9	0.95	8.7	0.96
	Rp-8-pCPT-2′-O-Me-cAMPS	>300	–	>300	–
	8-pCPT-6-Phe-cAMP	6.2	0.98	3.0	–
	8-pCPT-6-Phe-dcAMP	8.2	–	8.00	–
Br-substituted cAMP analogs	8-Br-cAMP	>300	–	>300	–
	Rp-8-Br-cAMPS	>300	–	>300	–
	8-Br-2′-O-Me-cAMP	>300	–	>300	–
Butyryl-substituted cAMP analogs	6-MB-cAMP	>300	–	>300	–
	DB-cAMP	>300	–	119	0.64
cGMP analogs	cGMP	>300	–	>300	–
	8-pCPT-cGMP	259	0.98	88.8	0.98
	8-Br-cGMP	>300	–	>300	–
Adenosine analogs	Adenosine	>300	–	>300	–
	8-pCPT-Ado	>300	–	150	–
	8-pCPT-2′-O-Me-Ado	31.3	0.98	17.2	0.82

1 : HEK293 cells transfected with either OATP1B1 or OATP1B3 were incubated with increasing doses of cAMP analogs for 15 min before addition of Nod (0.1 µM for OATP1B1 and 0.6 µM for OATP1B3). After 90 min coincubation, the cells were fixed in 2% buffered formaldehyde, and percent apoptotic cells determined by microscopic evaluation of surface membrane. The data was used to estimate IC50 values using a four parameter curve fitting (see Experimental section). The panels show typical data for cAMP analogs inhibiting apoptosis (left) or analogs that do not interfere with apoptosis (right). All experiments were performed in triplicates or more. The R^2^ for each regression analysis is shown.

To explore whether the aromatic phenyl functionality could be the pivotal factor for cAMP-analogs to inhibit OATP1B1/1B3, we tested analogs with two such moieties. The analog 8-pCPT-6-Phe-cAMP and its 2′-deoxy variant were very potent inhibitors of both channels, with IC50 values well below 10 µM. This also provided even stronger evidence for lack of involvement of Epac activation, since the 6-substitution eliminate any activitory effect against Epac [1].

We next wanted to know if other commonly used substituents in cAMP analogs affected their ability to inhibit OATP1B1/1B3. Neither the 8-Br nor the 6-MB substitutions affected OATP1B1/1B3 transport. The addition of 2′-O-Me to 8-Br-cAMP did not affect OATP1B1 activity, but slightly inhibited OATP1B3. In line with this, addition of a 2′-O-butyryl substituent to 6-MB cAMP, to produce DB-cAMP, also led to weak inhibition of OATP1B3 activity, but not to OATP1B1 activity.

Finally, we tested if other nucleotide/nucleoside analogs could inhibit the liver transporters. Again, we found that the chemical introduction of an 8-pCPT substitution to either cGMP or adenosine (Ado) rendered them able to inhibit OATP-mediated Nod-transport, whereas for instance 8-Br-cGMP was inactive. We also noted that 8-pCPT-2′-O-Me-Ado was far more potent than 8-pCPT-cAMP, in line with the results for the respective cAMP analogs.

**Figure 5:**
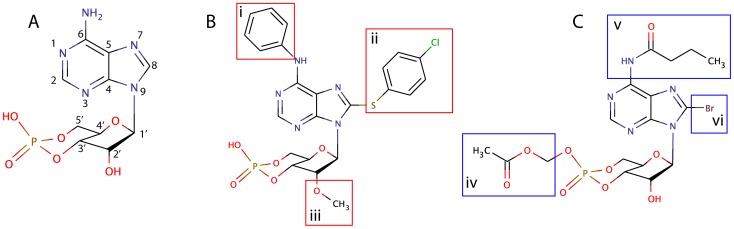
Chemical structures of A: cAMP, B: cAMP with substituents that favor inhibition of OATP1B1 and 1B3, and C: substituents that do not interfere with OATP-activity. The structure in A shows the numbering of the atoms. The substituents in B and C are i: N^6^-phenyl, ii: 8-*para*-chlorophenylthio, iii: 2′-O-methyl, iv: acetoxymethyl ester, v: N^6^-monobutyryl, vi: 8-bromo.

Taken together, the results in Table 1 indicate that the substituents 8-pCPT, 6-Phe and 2′-O-Me favor inhibitory properties of cyclic nucleotide analogs towards OATP1B1/1B3 (Fig. 5B) whereas the AM-esterification, 8-Br- and 6-MB substituents do not affect OATP1B1/1B3 activity (Fig. 5C). Native cAMP, cGMP or Ado did not affect OATP1B1/1B3 mediated transport of Nod into HEK293 cells (Table 1).

## Discussion

Based on the results presented, we conclude that cAMP analogs must be used with care when working with cells expressing bile acid carriers. We have identified three substituents that appear to interfere with OATP1B1/1B3 transport (Fig. 5B); first the 8-*para*-Chlorophenylthio (8-pCPT) group, which is common in several analogs selective to Epac or PKA, or used to mimic elevated cAMP levels. Secondly, the 2′-O-Me substituent, which led to increased potency of both 8-pCPT-cAMP and 8-pCPT-Ado (Table 1). It also caused some inhibition of the Epac-selective analog 8-Br-2′-O-Me-cAMP against OATP1B3, but not 1B1 (Table 1). The third substituent affecting OATP-activity is the 6-Phe group. The addition of this aromatic moiety to 8-pCPT-cAMP led to a strongly increased potency of 10-fold for OATP1B3, and 20-fold for OATP1B1. While these analogs are reported to be potent PKA- or Epac1 agonists [1], [42], the almost inactive 2′-deoxy-variant was equally active in our study (Table 1), supporting that inhibition of Nod-induced apoptosis is PKA and Epac independent.

Neither 8-Br, 6-MB, nor the AM-esterification affected Nod-mediated transport (Figure 2, Table 1). It thus appears that the addition of a bulky substituent at the N^6^-position is no prerequisite for OATP-inhibition, while the presence of an aromatic ring substituent is (Table 1 and Fig. 5B and C), pinpointing to potential charge-transfer interactions with aromatic amino acids of the channels. In line with this, we have found that the cyclic pentapeptide Nostocyclopeptide-M1 containing three aromatic tyrosine residues was a more potent inhibitor of OATP1B1/1B3 than either of the cAMP analogs tested here [20].

The interior of the transmembrane cylinders of OATP1B1 and 1B3 contain several aromatic amino acids [43] that can contribute to π-π interactions [44], [45], and positively charged Lys and Arg [46], [47], which can form strong cation-π interactions with aromatic parts of the ligand [48] (see Fig. S3). Several studies have identified aromatic or cationic amino acids as important in OATP1B1/1B3 mediated transport [43], [49]. This could explain why an increasing amount of aromatic substituents on a substrate increased its ability to compete with natural substrates of OATP1B1 and 1B3. Recent studies showed that general inhibitors of OATP1B1 and 1B3 all had several aromatic ring structures [39], [50], as was the case with the most potent inhibitors in the present study (Fig. 5B). However, the less studied and structurally flexible extracellular loops of OATP1B1/1B3 could also be regions for molecular interactions with cAMP analogs.

The analogs with high ability to inhibit OATP1B1/1B3 mediated transport are highly hydrophobic (logD, Fig. S4A and B), which is in concordance with previous findings on OATP1B1/1B3 inhibitors [50]. One could believe that their increased ability to cross biological membranes [51] may favor intracellular effect, but the even more permeable AM-ester of 8-pCPT-2′-O-Me-cAMP did not affect hepatocyte death or [^3^H]-Nod accumulation (Fig. 2A and B), suggesting that the effect was not mediated through the inside of the cells. It appears that the presence of aromatic substituents is the major determining factor in our study (Tab. 1 and Fig. 5). A surprising finding was the dramatic difference between the *S*p and *R*p configuration of 8-pCPT-2′O-Me-cAMPS on the ability to inhibit Nod-induced apoptosis in OATP1B1/1B3 transfected HEK293 cells (Table 1). A dihedral scan did not reveal different preferences towards syn and anti configuration between the *R*p and *S*p configuration of 8-pCPT-2′O-Me-cAMPS (Fig. S5), and the mechanisms behind this discrepancy towards OATP-inhibition remains enigmatic.

Off-target effects of cyclic nucleotides have been reported in other systems, such as inhibition of platelets [6], [7], [52]. The pCPT-substituents at the 8-position have been identified as relevant for the off-target effects on Equilibrative Nucleoside Transporter 1 and activation of A2A Adenosine Receptors in nucleated cells [53]. In our system, we show that other substituents, such as 6-Phe and 2′-O-Me can additionally exhibit or enhance off-target effects of cyclic nucleotides on OATP1B1 and 1B3.

The off-target inhibition of OATP1B1/1B3 by cyclic nucleotide analogs is likely to be present in freshly isolated primary hepatocytes or cell systems with enforced expression of these channels. It has been shown that the expression of these channels is significantly reduced in primary hepatocytes only 6 hours after isolation [54]. However, several cancers [23], [55], [56], [57] and cancer cell lines [24], [25] have increased expression of OATP1B3, and this must be taken into consideration when working with cAMP and cGMP analogs in non-primary cell systems. In such systems, also analogs without aromatic ring substituents [1], [2] and with little or no effect on the OATP channels (Table 1) should be tested before concluding that an effect is mediated via PKA or Epac.

## Supporting Information

Figure S1 **Elevation of intracellular cAMP in HEK293T cells after stimulation by forskolin and IBMX.** HEK293T cells were added forskolin (50 µM) and IBMX (250 µM) and processed for measurement of intracellular cAMP as described Jensen et al. (Platelets 2011, 22, 8–19).
(TIF)

Figure S2 **Inverse relationship between dose and accumulation of [^14^C]-glycocholic acid in hepatocytes.** Rat hepatocytes were incubated with the given concentrations of cAMP analogs for 15 min before addition of [^14^C]-glycocholic acid. After 15 min, the cells were separated from the medium and radioactivity measured by liquid scintillation. Note that while low concentrations (50 µM) of 8-pCPT-cAMP or 6-MB-cAMP cause increased uptake of [^14^C]-glycocholic acid, higher concentrations have less or no effect.
(TIF)

Figure S3 **A computer-generated model of OATP1B1 shows several residues able to interact with aromatic substituents on cAMP.** Visualization of amino acids in the transmembrane cylinder of OATP1B1, likely to interact with aromatic parts of the cAMP analogs by either π-π interactions (green) or cation-π interactions (red). The transmembrane model of OATP1B1 was generated by the protein structure prediction service Phyre2 (http://www.imperial.ac.uk/phyre/). Note that this is a tentative model that lacks extra- and intracellular loops that could be crucial in substrate recognition, and that only minor alterations in 3D-structure can change amino acid orientation. However, based on mutation studies (Li, N. et al. PloS ONE 2012, 7, e36647 and Gui, C. and Hagenbuch, B. Biochemistry 2008, 47, 9090-7.), several of the depicted amino acids are important for substrate recognition and transport. Visualization of the protein model was by Discovery Studio 3.1 (2011, accelrys software, www.accelrys.com).
(TIF)

Figure S4 **Ability to inhibit OATP1B1/1B3 mediated uptake correlates with lipophilicity of the substituent on the adenosine part, but not on the cyclic phosphate of cAMP.** The IC50 data (Table 1 in the original paper) was divided by 100, and plotted against LogD. Log D was estimated with the Calculator Plug-in in Marvin version 5.7 (2012) for Apple (ChemAxon Ltd., www.chemaxon.com). The molecules were drawn in 2D mode and converted to 3D structures with the fine build clean 3D mode in Marvin. Standard ionic condition (0.1 M Cl- and 0.1 M Na+/K+) was used for estimation of logD at pH (7.4).
(TIF)

Figure S5 **Dihedral scan of cAMP analogs reveal two preferred conformations.** Gaussian 09 was used to calculate the energy profile as a function of dihedral angle connecting the two cAMP ring systems. The dihedral angle was incremented by 10 degrees with geometry optimization at the HF/6-31G* level of theory using the Gaussian 09 (Rev. b.01) software (Gaussian, Inc., Wallingford CT, 2009). The inserted structures show syn and anti configuration.
(TIF)

Table S1 Based on the determined IC50 values (Table 1) we have estimated the competitive inhibitor constant, K_i_, of the tested cyclic nucleotide analogs for OATP1B1 and OATP1B3. Unlike IC50, this constant is not similarly dependent on experimental conditions like toxin concentration and the K_m_ value for Nod of OATP1B1 and OATP1B3. See the Methods section and (Herfindal et al. Mol Pharm. 2011, 8, 360-7) for details concerning estimation of constants.
(DOC)
