# Supplementary figures and images for: Introduction of Aromatic Ring-Containing Substituents in Cyclic Nucleotides Is Associated with Inhibition of Toxin Uptake by the Hepatocyte Transporters OATP 1B1 and 1B3
Source: PLoS One. 2014 Apr 16;9(4):e94926. doi: 10.1371/journal.pone.0094926 (PMC3989234; doi:10.1371/journal.pone.0094926)

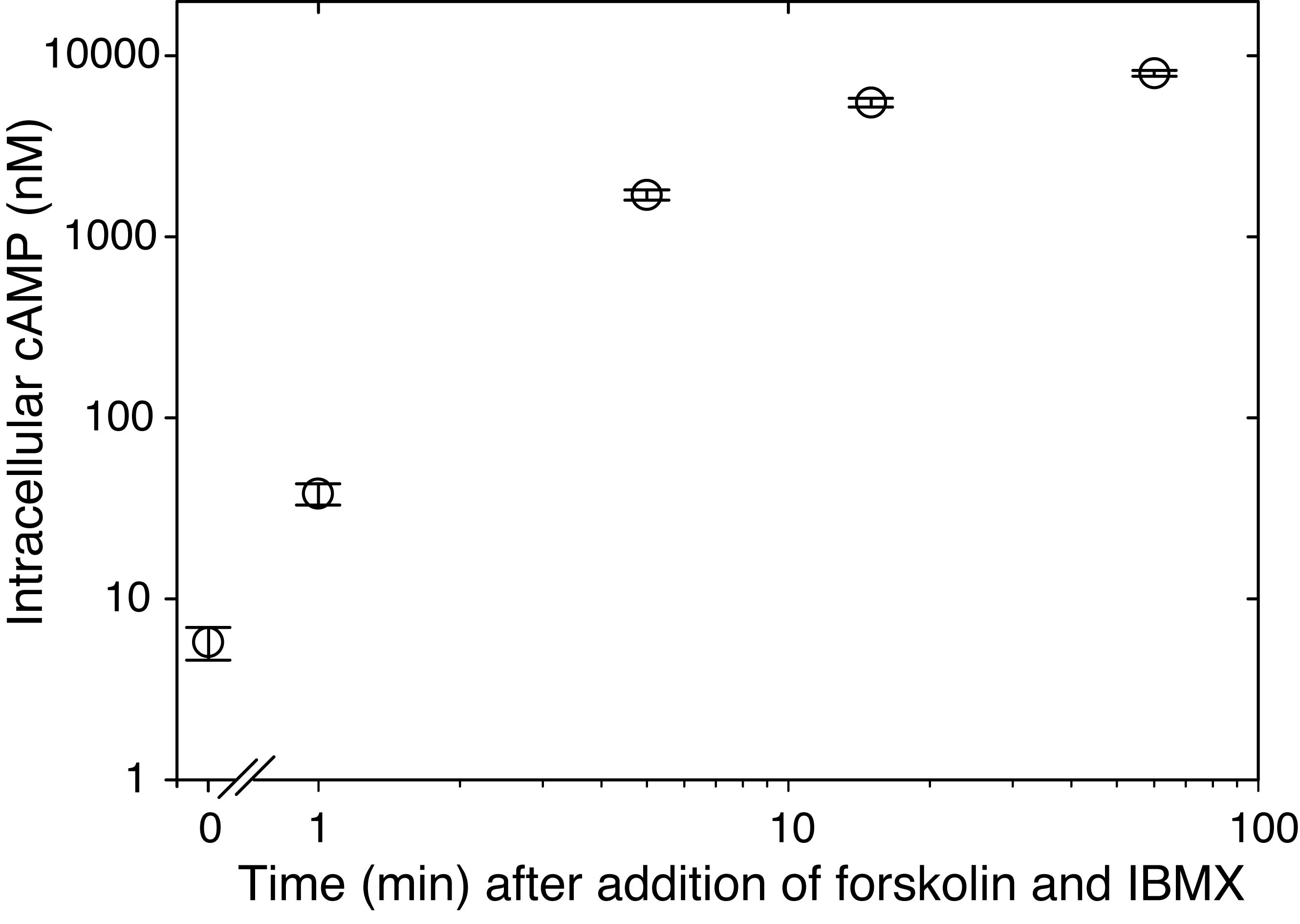

Supplement: Figure S1 — Elevation of intracellular cAMP in HEK293T cells after stimulation by forskolin and IBMX. HEK293T cells were added forskolin (50 µM) and IBMX (250 µM) and processed for measurement of intracellular cAMP as described Jensen et al. (Platelets 2011, 22, 8–19). (TIF) [file pone.0094926.s001.tif]

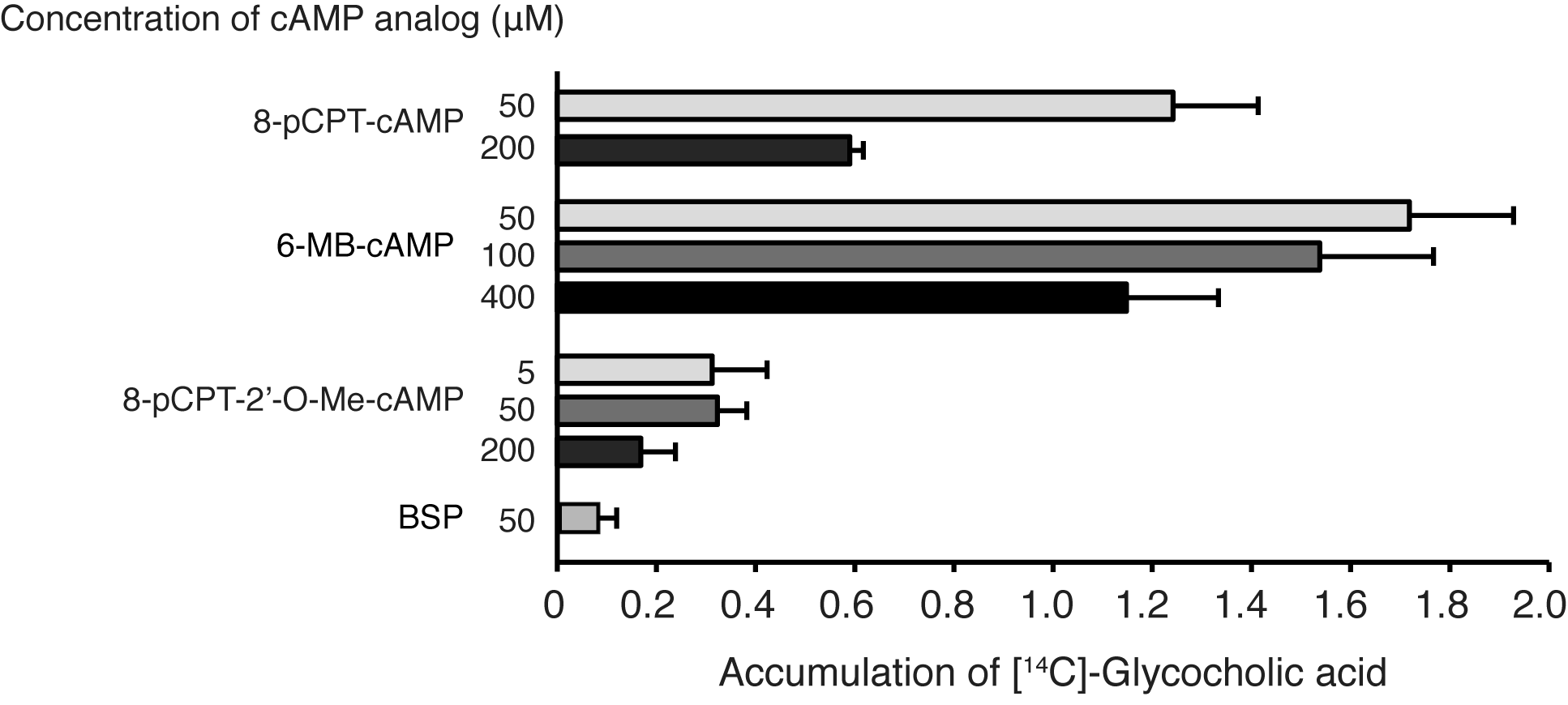

Supplement: Figure S2 — Inverse relationship between dose and accumulation of [14C]-glycocholic acid in hepatocytes. Rat hepatocytes were incubated with the given concentrations of cAMP analogs for 15 min before addition of [14C]-glycocholic acid. After 15 min, the cells were separated from the medium and radioactivity measured by liquid scintillation. Note that while low concentrations (50 µM) of 8-pCPT-cAMP or 6-MB-cAMP cause increased uptake of [14C]-glycocholic acid, higher concentrations have less or no effect. (TIF) [file pone.0094926.s002.tif]

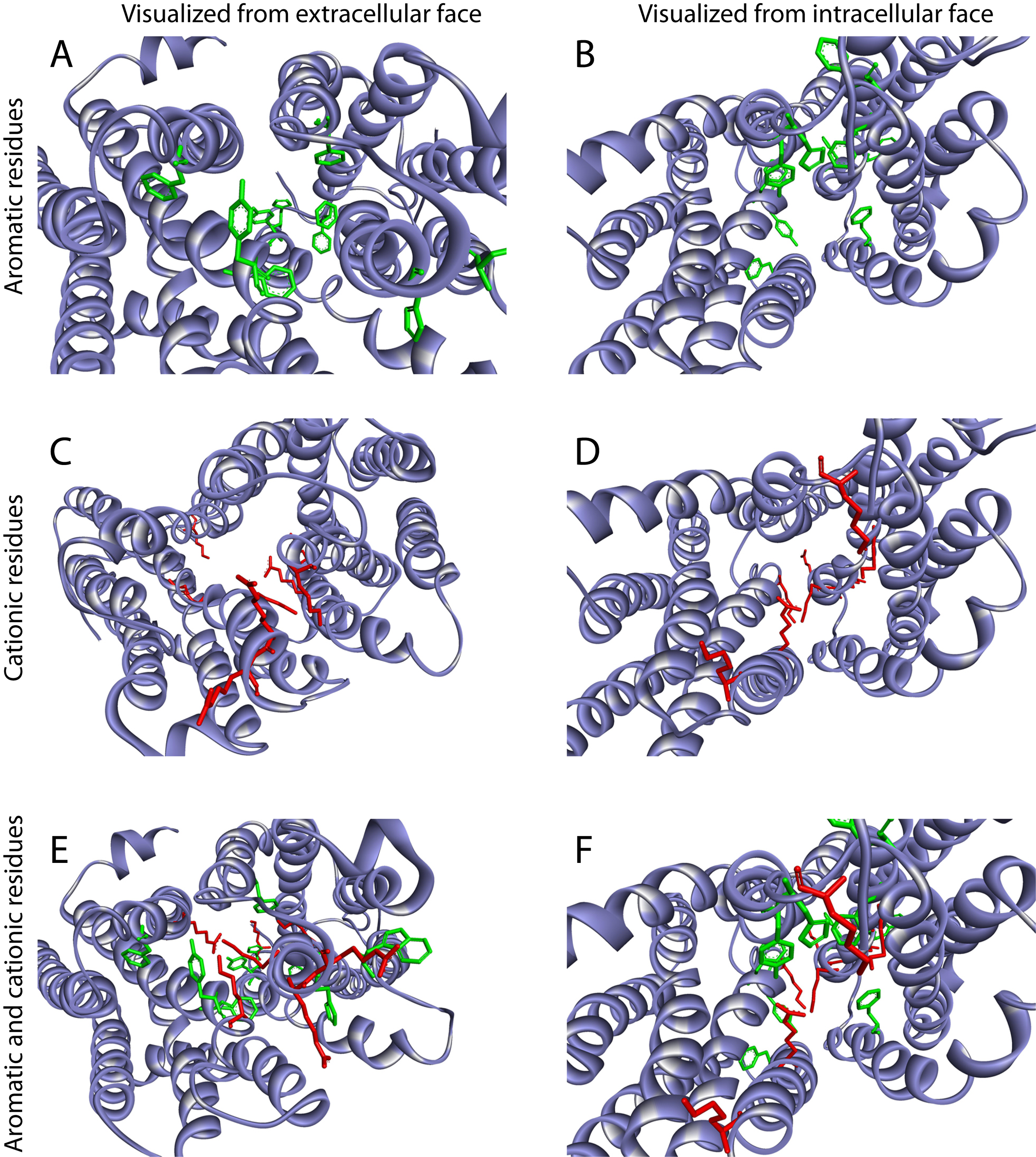

Supplement: Figure S3 — A computer-generated model of OATP1B1 shows several residues able to interact with aromatic substituents on cAMP. Visualization of amino acids in the transmembrane cylinder of OATP1B1, likely to interact with aromatic parts of the cAMP analogs by either π-π interactions (green) or cation-π interactions (red). The transmembrane model of OATP1B1 was generated by the protein structure prediction service Phyre2 (http://www.imperial.ac.uk/phyre/). Note that this is a tentative model that lacks extra- and intracellular loops that could be crucial in substrate recognition, and that only minor alterations in 3D-structure can change amino acid orientation. However, based on mutation studies (Li, N. et al. PloS ONE 2012, 7, e36647 and Gui, C. and Hagenbuch, B. Biochemistry 2008, 47, 9090-7.), several of the depicted amino acids are important for substrate recognition and transport. Visualization of the protein model was by Discovery Studio 3.1 (2011, accelrys software, www.accelrys.com). (TIF) [file pone.0094926.s003.tif]

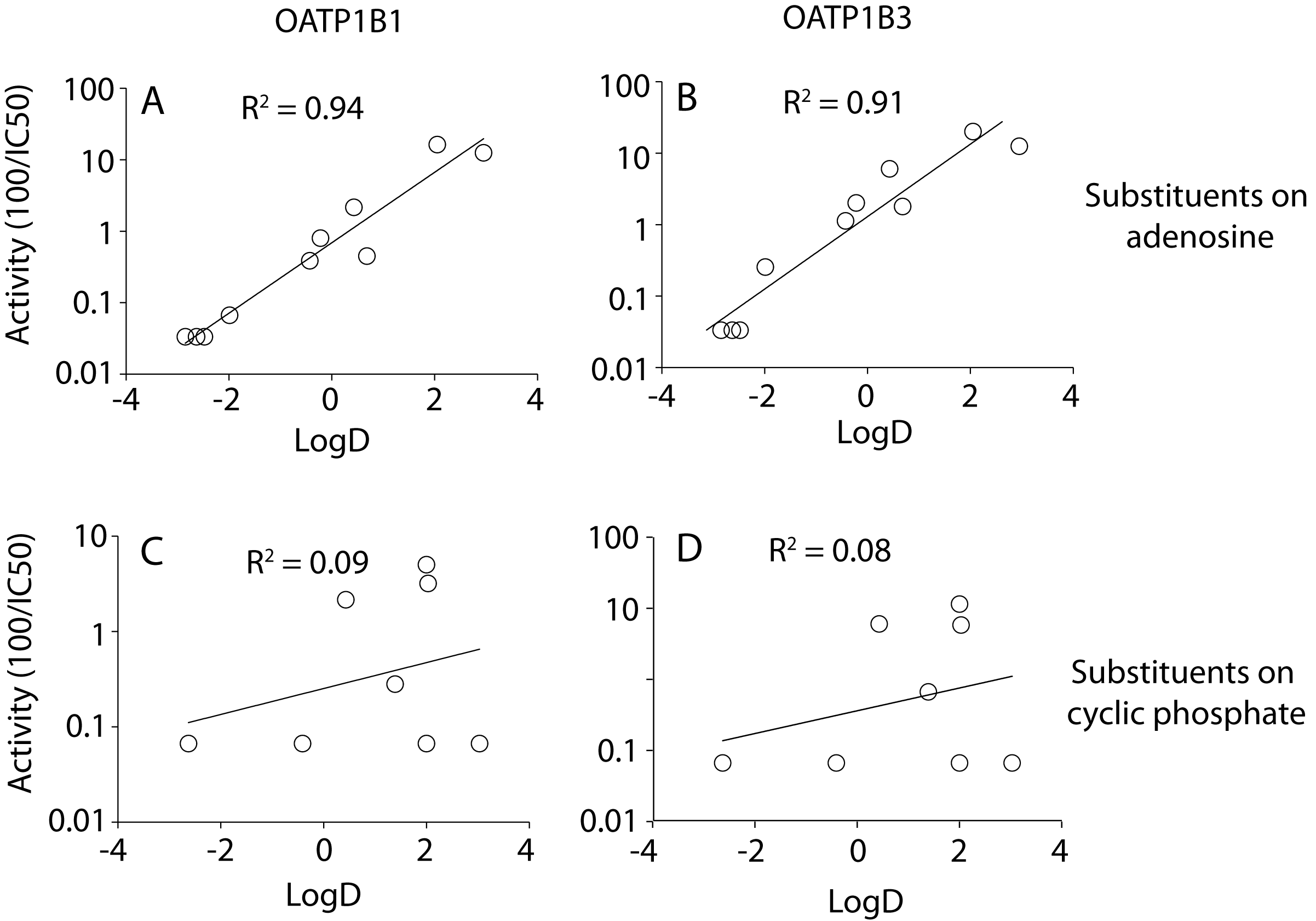

Supplement: Figure S4 — Ability to inhibit OATP1B1/1B3 mediated uptake correlates with lipophilicity of the substituent on the adenosine part, but not on the cyclic phosphate of cAMP. The IC50 data (Table 1 in the original paper) was divided by 100, and plotted against LogD. Log D was estimated with the Calculator Plug-in in Marvin version 5.7 (2012) for Apple (ChemAxon Ltd., www.chemaxon.com). The molecules were drawn in 2D mode and converted to 3D structures with the fine build clean 3D mode in Marvin. Standard ionic condition (0.1 M Cl- and 0.1 M Na+/K+) was used for estimation of logD at pH (7.4). (TIF) [file pone.0094926.s004.tif]

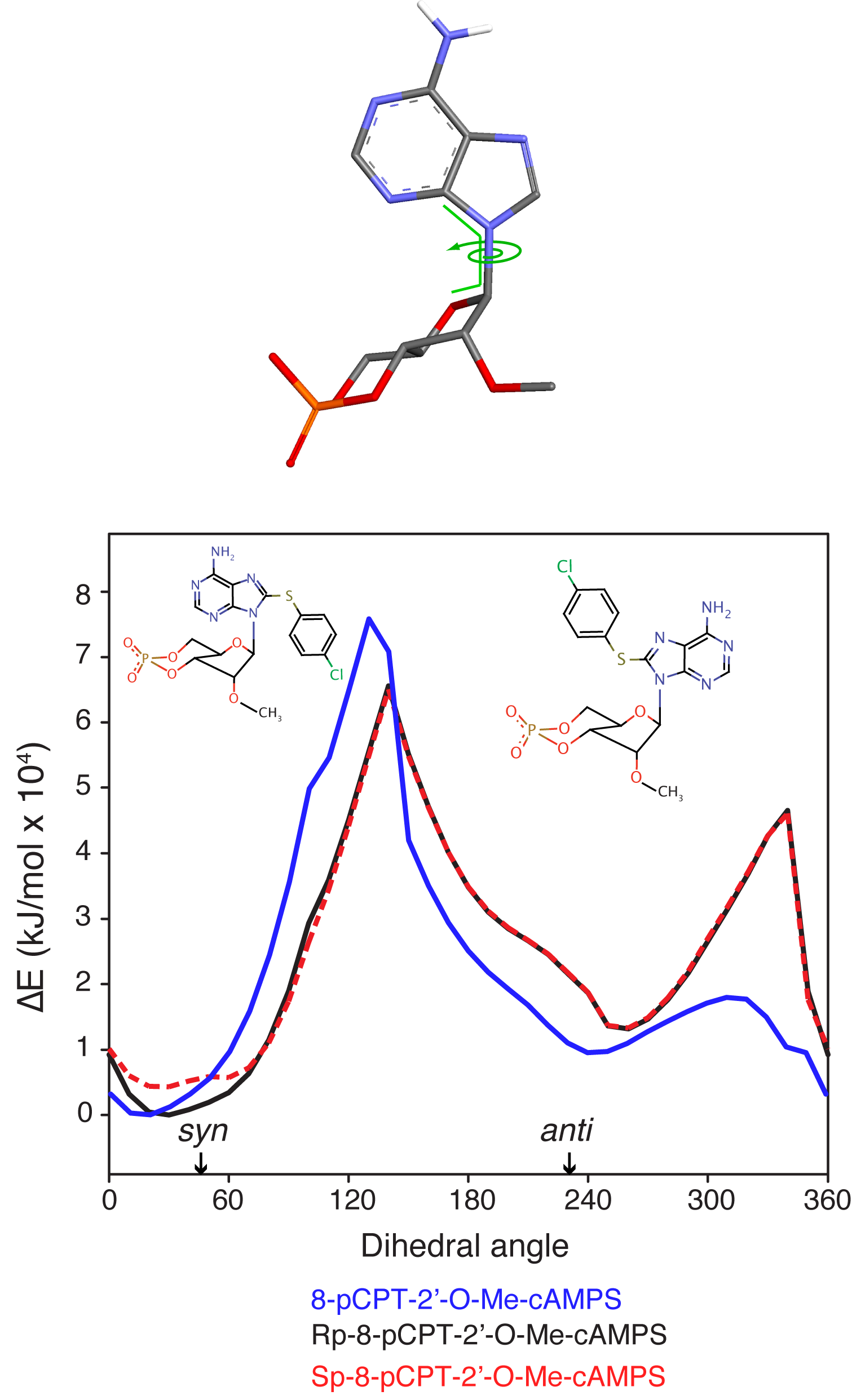

Supplement: Figure S5 — Dihedral scan of cAMP analogs reveal two preferred conformations. Gaussian 09 was used to calculate the energy profile as a function of dihedral angle connecting the two cAMP ring systems. The dihedral angle was incremented by 10 degrees with geometry optimization at the HF/6-31G* level of theory using the Gaussian 09 (Rev. b.01) software (Gaussian, Inc., Wallingford CT, 2009). The inserted structures show syn and anti configuration. (TIF) [file pone.0094926.s005.tif]
