# Supplementary material for: Introduction of Aromatic Ring-Containing Substituents in Cyclic Nucleotides Is Associated with Inhibition of Toxin Uptake by the Hepatocyte Transporters OATP 1B1 and 1B3
Source: PLoS One. 2014 Apr 16;9(4):e94926. doi: 10.1371/journal.pone.0094926 (PMC3989234; doi:10.1371/journal.pone.0094926)
Supplement: Table S1 — Based on the determined IC50 values (Table 1) we have estimated the competitive inhibitor constant, Ki, of the tested cyclic nucleotide analogs for OATP1B1 and OATP1B3. Unlike IC50, this constant is not similarly dependent on experimental conditions like toxin concentration and the Km value for Nod of OATP1B1 and OATP1B3. See the Methods section and (Herfindal et al. Mol Pharm. 2011, 8, 360-7) for details concerning estimation of constants. (DOC) [file pone.0094926.s006.doc]

|  | OATP1B1 | | | OATP1B3 | | |
| --- | --- | --- | --- | --- | --- | --- |
| Analog | IC50  (nM) | R^2^ | K_i_^a^  (nM) | IC50  (nM) | R^2^ | K_i_^a^  (nM) |
| pCPT-containing cAMP analogs |  |  |  |  |  |  |
| 8-pCPT-cAMP | 130 | 0.98 | 43.3 | 49.6 | 0.98 | 13.3 |
| 8-pCPT-dcAMP | 223 | 0.94 | 74.7 | 55.7 | 0.98 | 14.9 |
| 8-pCPT-2’-O-Me-cAMP | 46.4 | 0.98 | 15.5 | 12.6 | 0.96 | 3.38 |
| Sp-8-pCPT-2’-O-Me-cAMPS | 19.9 | 0.95 | 6.63 | 8.7 | 0.96 | 2.33 |
| Rp-8-pCPT-2’-O-Me-cAMPS | >300 | – | - | >300 | – | - |
| 8-pCPT-6-Phe-cAMP | 6.2 | 0.98 | 2.07 | 3.0 | – | 0.804 |
| 8-pCPT-6-Phe-dcAMP | 8.2 | – | 2.73 | 8.00 | – | 2.14 |
| Br-containing cAMP analogs |  |  |  |  |  |  |
| 8-Br-cAMP | >300 | – | - | >300 | – | - |
| Rp-8-Br-cAMPS | >300 | – | - | >300 | – | - |
| 8-Br-2’-O-Me-cAMP | >300 | – | - | >300 | – | - |
| MB-cAMP analog |  |  |  |  |  |  |
| 6-MB-cAMP | >300 | – | - | >300 | – | - |
| cGMP analogs |  |  |  |  |  |  |
| 8-pCPT-cGMP | 259 | 0.98 | 86.3 | 88.8 | 0.98 | 29.6 |
| 8-Br-cGMP | >300 | – | - | >300 | – | - |
| Adenosine analogs |  |  |  |  |  |  |
| 8-pCPT-Ado | >300 | – | - | 150 | – | 40.2 |
| 8-pCPT-2’-O-Me-Ado | 31.3 | 0.98 | 10.4 | 17.2 | 0.82 | 4.61 |

^a^Estimated K_i_ values are calculated assuming conditions for competitive inhibition are fulfilled, using equation 2 and K_m_ values for Nod of 50 and 220 nM for OATP1B1 and OATP1B3, respectively.
